# In Xenopus ependymal cilia drive embryonic CSF circulation and brain development independently of cardiac pulsatile forces

**DOI:** 10.1186/s12987-020-00234-z

**Published:** 2020-12-11

**Authors:** A. H. Dur, T. Tang, S. Viviano, A. Sekuri, H. R. Willsey, H. D. Tagare, K. T. Kahle, E. Deniz

**Affiliations:** 1 Department of Pediatrics, Yale University School of Medicine, 333 Cedar Street, New Haven, CT 06510 USA; 2 Acibadem Mehmet Ali Aydinlar University School of Medicine, Istanbul, Turkey; 3 Department of Radiology and Biomedical Imaging, Yale University, 300 Cedar St, New Haven, CT 06510 USA; 4 Department of Psychiatry and Behavioral Sciences, UCSF Weill Institute for Neurosciences, University of California, San Francisco, San Francisco, CA 94143 USA; 5 Department of Neurosurgery and Cellular & Molecular Physiology, and Centers for Mendelian Genomics, Yale University School of Medicine, 333 Cedar Street, New Haven, CT 06510 USA; 6 Pediatric Genomics Discovery Program, Yale University School of Medicine, 333 Cedar Street, New Haven, CT 06510 USA

**Keywords:** Congenital hydrocephalus, Ependymal cilia, Optical coherence tomography, Xenopus tropicalis, Embryonic CSF circulation

## Abstract

**Background:**

Hydrocephalus, the pathological expansion of the cerebrospinal fluid (CSF)-filled cerebral ventricles, is a common, deadly disease. In the adult, cardiac and respiratory forces are the main drivers of CSF flow within the brain ventricular system to remove waste and deliver nutrients. In contrast, the mechanics and functions of CSF circulation in the embryonic brain are poorly understood. This is primarily due to the lack of model systems and imaging technology to study these early time points. Here, we studied embryos of the vertebrate *Xenopus* with optical coherence tomography (OCT) imaging to investigate in vivo ventricular and neural development during the onset of CSF circulation.

**Methods:**

Optical coherence tomography (OCT), a cross-sectional imaging modality, was used to study developing *Xenopus* tadpole brains and to dynamically detect in vivo ventricular morphology and CSF circulation in real-time, at micrometer resolution. The effects of immobilizing cilia and cardiac ablation were investigated.

**Results:**

In *Xenopus*, using OCT imaging, we demonstrated that ventriculogenesis can be tracked throughout development until the beginning of metamorphosis. We found that during *Xenopus* embryogenesis, initially, CSF fills the primitive ventricular space and remains static, followed by the initiation of the cilia driven CSF circulation where ependymal cilia create a polarized CSF flow. No pulsatile flow was detected throughout these tailbud and early tadpole stages. As development progressed, despite the emergence of the choroid plexus in *Xenopus*, cardiac forces did not contribute to the CSF circulation, and ciliary flow remained the driver of the intercompartmental bidirectional flow as well as the near-wall flow. We finally showed that cilia driven flow is crucial for proper rostral development and regulated the spatial neural cell organization.

**Conclusions:**

Our data support a paradigm in which *Xenopus* embryonic ventriculogenesis and rostral brain development are critically dependent on ependymal cilia-driven CSF flow currents that are generated independently of cardiac pulsatile forces. Our work suggests that the *Xenopus* ventricular system forms a complex cilia-driven CSF flow network which regulates neural cell organization. This work will redirect efforts to understand the molecular regulators of embryonic CSF flow by focusing attention on motile cilia rather than other forces relevant only to the adult.

## Background

Cerebrospinal fluid (CSF) provides hydromechanical protection to the central nervous system (CNS) and delivers nutrients and active metabolites, while also eliminating catabolites. Hydrocephalus develops when cerebral ventricles pathologically expand due to CSF accumulation within the ventricles, leading to a group of diseases with high mortality and morbidity [1, 2]. In adults, the bulk of the CSF flows from the choroid plexuses, with the pulsatile arterial flow acting as a pump to drive CSF movement through the ventricles [3, 4], in conjunction with respiratory movements [5]. Besides these properties, emerging data underscore the critical role CSF plays in early embryonic neurogenesis, where CSF maintains appropriate neuroprogenitor identity, proliferation, and differentiation [6–10]. Importantly, mutations in genes expressed predominantly by the neuroprogenitor cells have been shown to lead to congenital hydrocephalus [11]. These findings implicate a new embryonic perspective in hydrocephalus pathogenesis, suggesting that neuroprogenitors may contribute to a hydrocephalus outcome. In line with these data, the role of CSF circulation in regulating neurogenesis is emerging. For example, in the adult brain, neuroblasts born in the subventricular zone migrate from the walls of the lateral ventricles to the olfactory bulb, suggesting that CSF circulation directly regulates neuronal migration and axon pathfinding [12]. However, a specific mechanism of how ventricular embryonic CSF circulation is sensed and directs this process remains unknown. This is, in part, due to technical limitations in studying embryonic ventriculogenesis and CSF dynamics. Therefore, it is essential to understand what constitutes early CSF dynamics.

There are two significant drivers of the CSF circulation, the heart, and the ependymal cilia. The heart is known to drive the bulk CSF circulation. Flow, in this case, is generated by the expulsion of the CSF via dilated intracranial arteries during cardiac systole. Based on the magnetic resonance imaging and radionuclide cisternography studies in adult human brains, this bulk flow is driven mainly by the choroid plexus pulsations, and additionally by the ventricular wall motion [13]. When compared to the cilia-driven near-wall flow, heart-driven flow is thought to be several magnitudes higher [13–19]. In humans, CSF is mostly produced, secreted, and circulated by the choroid plexus, which appears on the 41st day of embryogenesis. However, before choroid plexus formation and active CSF secretion, the neural tube progressively dilates and forms the primitive fluid-filled ventricular space. The determinants or even the existence of an embryonic CSF circulation at these pre-choroid plexus stages remain unclear in mammals. In contrast to the adult data, zebrafish work showed that the cardiac pulsatility might partly drive embryonic CSF circulation. The early work by Fame et al. showed that the CSF movement was partially dependent on the heartbeat, where the authors noted changes in CSF movement velocities when the heart was stopped or analyzed in mutants with no heart beat [20]. Subsequent work in zebrafish by Olstad et al. revealed that CSF flow was confined within individual ventricular cavities and driven by the ependymal cilia, with little exchange of fluid between ventricles, despite a pulsatile CSF displacement caused by the heartbeat [21]. Additionally, Thouvenin et al. further demonstrated the presence of non-efficient pulsatile flow in the zebrafish ventricular system, which did not impact central canal formation nor bidirectional central canal CSF circulation [22]. These observations suggested that the heart and pulsatile CSF flow may only have a limited role in embryonic CSF circulation.

The second driver of CSF flow is the cilia. These organelles are composed of a microtubule-based cytoskeleton that protrudes from the apical pole of the cell membrane and are connected to the cytoplasm through the basal body. The cilia have diverse sensory, motility, and signaling functions, which play an essential role in many distinct cellular functions, including motion, cell division, mechanosensation, body axis formation, and extracellular fluid flow generation [23]. Virtually all neurons are ciliated [24]. There are two types of ciliated cells in the CNS—multiciliated cells (MCCs) and monociliated cells. MCCs are found in the CNS, including the choroid plexus [25, 26]. Numerous cilia arise from the apical membrane of MCCs, protruding extracellularly up to ~ 20 µm, and beat unidirectionally, creating a planar polarized CSF circulation [27–29]. In the case of monociliated cells, single cilia can be either immotile or motile [30]. Specifically, immotile monocilia are referred to as primary cilia in the CNS, and numerous studies implicate their sensory role in sonic hedgehog signaling, Platelet-Derived Growth Factor (PDGF) signaling and Wnt signaling [31]. Recent work by Faubel et al. showed a network of streams driven by MCCs along the lateral ventricle of mouse suggesting the existence of a complex polarization along the ventricular surfaces [32]. The significance of this organization remains to be a daunting puzzle yet it is known that functional cilia are crucial for early brain development [33] (mouse, rat, pig [32], zebrafish [21], *Xenopus* [34], human [35]). Overall, the role of cardiac and ciliary forces in CSF circulation continues to be an area of intense research although limited by the availability of model systems and technical difficulties accessing the embryonic CNS in vivo.

*Xenopus* tadpole brains, although architecturally simpler, share the distinct compartmentalization of the mammalian brain with two lateral ventricles, third and fourth ventricle, but have the additional advantage of being semi-transparent during development. We have previously exploited this feature to demonstrate that the developing *Xenopus* brain is ciliated and to demonstrate a genetic hydrocephalus model with established hydrocephaly genes [34]. Here, we use this model to test the role of cardiac and ciliary forces on *Xenopus embryonic* CSF circulation as well as their role in *Xenopus* neurodevelopment.

Optical Coherence Tomography (OCT), is a cross-sectional imaging modality that dynamically detects in vivo ventricular morphology and CSF circulation in real-time, at micrometer resolution [34, 36–39]. Using OCT imaging, in this work we have formed an in vivo map of the *Xenopus* ventricular system from immediately after neurulation to the late tadpole stage when four ventricles are morphologically distinct. In real-time, we were able to observe the rostral to caudal expansion of the ventricles and determine the timing of the initiation of the CSF circulation. We show that embryonic CSF flow is initiated ~ 12 h post-neurulation (incubation at 25 °C—Stage 32). We have noted that CSF circulation becomes polarized and accelerated over time, with the circulatory flow being tenfold faster at the caudal region when compared to the rostrum. Based on prior observations in zebrafish [20, 21] and adult humans [13], we assumed that this circulation in *Xenopus* was generated mostly by ciliary and partially by the pulsatile cardiac forces. To tease out the compartmental contribution of cardiac forces, we removed the heart by microdissection. We detected no disturbance in CSF flow pattern, speed, or any change in the ventricular size or shape. When we paralyzed cilia genetically or with drugs, we did not appreciate any pulsatile flow throughout the tailbud or early tadpole stages. The only weak pulsatile flow was detected later in development around the hypothalamic region, which did not contribute to overall CSF circulation. The CSF continued to circulate and mix in all ventricles. Ependymal cilia sustained bidirectional flow across the ventricles via narrow channels. Lastly, we showed that in *Xenopus* rostral neuroprogenitor organization relies on cilia-driven CSF flow. Following the loss of cardiac circulation, as expected, these tadpoles developed body edema but intriguingly, retained not only CSF circulation but also spatial neuroprogenitor cell organization. In contrast, when we paralyzed cilia, we detected no CSF circulation, a significantly malformed CNS, and loss of compartmental organization of the neuroprogenitor cells.

In summary, our work shows that in *Xenopus,* the early embryonic ventricular system has already formed an independent ependymal cilia-driven polarized CSF flow network that drives the CSF within and across the ventricular space. This network regulates polarized intraventricular CSF circulation, interventricular CSF mixing, and compartmental neural cell organization. During the early embryogenesis, we did not detect any pulsatile CSF flow in *Xenopus*. These findings suggest that during embryogenesis, *Xenopus* embryonic CSF circulatory system undergoes two phases, where the primitive ventricular space is initially filled with pre-choroidal CSF and remains static. Followed by the second phase when the cilia drive pre-choroidal CSF circulation where polarized ependymal cilia create a polarized flow, which in part regulates spatial neural cell organization.

## Materials and methods

### Xenopus husbandry

*Xenopus tropicalis* were housed and cared for in our aquatics facility according to established protocols approved by Yale Institutional Animal Care and Use Committee (IACUC).

### Microinjections

In vitro fertilization and microinjections were conducted as previously described [40] and standard protocols [41]. Embryos were injected at the one-cell stage. For microinjections, borosilicate glass needles calibrated to inject 10 ng *c21orf59* morpholino oligonucleotide [42] (5′-CCTTCTTAACGTGTAAGCGCACCAT-3′, Gene Tools, LLC) with Dextran Alexa 488 tracer (Invitrogen). After injections, the embryos were left in 1/9 × MR + 3% Ficoll for 1 h at room temperature and then transferred to 1/9X MR supplemented with 0.1% gentamycin and incubated at 26 °C. Injections were confirmed with fluorescent lineage tracing with a Zeiss Lumar fluorescence stereomicroscope at stage 20 and embryos were left to develop at 26 °C until stage 46.

### Optic coherence tomography imaging

We used a Thorlabs Ganymede 900 nm spectral domain-OCT Imaging System, which allows for 1.4 mm imaging depth in the air + water − 2.2 μm Axial Resolution in water and lateral resolution 4 µm. We obtained 2D cross-sectional images and measurements were obtained with ThorImageOCT software version 4.4.6.

- Ventricular developmental map:
  We serially imaged *Xenopus* embryos using OCT, starting at stage 16, continuing to stage 21 in 1/9 × MR. To image the same embryos from stage 22–48, they were embedded in 1% low melt agarose (AmericanBio) in 1/9 × MR to immobilize them mechanically. To embed the embryos, the low melt agarose solution was heated until the agarose was completely dissolved, transferred to a dish, then allowed to cool slightly. Embryos were then transferred to the dish and oriented with the dorsal side facing the OCT imaging field. The agarose was then allowed to solidify for 3–4 min. Following image acquisition, tadpoles removed from the agar for recovery and re-embedded at the next stage of development using developmental markers outlined by Nieuwkoop and Faber [NF] staging [43].
- CSF flow velocity analysis and particle tracking:
  Particle tracking was performed using TrackMate particle tracker plugin built in ImageJ. The plugin allows two-dimensional tracking and analysis of particle trajectories. The region of interest (ROI), the ventricle, was segmented manually. We obtained a velocity color map and a vector map that shows all five flow fields. We also obtained median velocities (described below in detail) from each flow field (lateral, 3rd, midbrain, and 4th ventricle), and we compared experimental velocities with the corresponding ventricle velocities in controls.
- Gaussian process regression:
  We used a Gaussian process regression (GPR)—based post-processing ensemble method, reported in [44], to accurately estimate fluid velocity in a dense user-selected region. The method calculates dense velocity estimates via a three-stage process.
  Stage 1 uses the LAP simple tracking algorithm provided by TrackMate [45–47] to obtain noisy, sparse ‘tracks’ from OCT image sequences. These tracks indicate the presence and trajectory of particles identified by the TrackMate algorithm. Stage 2 partitions each trajectory into a small number of frames, which is roughly equivalent to a half-second or full-second of data. For each partitioned section of a trajectory, a weighted least-squares fitting was used to calculate a velocity vector representing the movement of the particle during the partitioned time frame. The velocity vectors are reported as median velocities [44]. The output of Stage 2 is a set of velocity vectors sparsely scattered on the user-segmented region, and by design robust against noise or tracker irregularities. Stage 3 applies GPR with a modified version of Matlab’s fitrgp function (MATLAB, version 9.4.0 (R2018a)) (The MathWorks Inc., Natick, Massachusetts, 2018) to obtain a set of densely located velocity vectors that are spatially smooth and robust against data irregularities. GPR also gives an estimate of the uncertainty in the smoothed velocity vectors, the uncertainty being due to noise in the data. The uncertainty reported as a standard deviation of the estimated vectors (For further details about the algorithm, the reader is referred to our previous report [44]).
  A custom-made GUI, written in Matlab, visualizes the calculated dense fluid velocities, their standard deviations, and other statistics. The estimated velocity vector at each pixel is viewed as a quiver-map overlaid on top of an OCT image. The GUI also displays the speed (magnitude of the estimated velocity) and the standard deviation of the estimate as color-maps. Finally, the GUI allows selections of subregions (for example, the head of the *Xenopus*) to compute statistics for the subregion. To calculate the median speed in a subregion, for example, the software collects all of the vectors in the subregion and computes the median norm of the collected vectors, thus giving the median speed of movement in the subregion. The code for our GUI, including the entire three-stage GPR estimation pipeline, is available at https://github.com/tommymtang/PTVProcessor

### Heart ablation at stage 46

We used 35 × 10 mm petri dishes, each with a 10 mm diameter hole cut out of the bottom. An 18 mm glass coverslip was attached to the bottom of the dish covering the hole using vacuum grease to make a watertight seal. Stage 46 control tadpoles were embedded as described above into these dishes with 1.5 ml of 1% low melt agarose in 1/9× MR. They were embedded with the dorsal side facing the light source and the ventral side close to the glass coverslip covering the opening at the bottom of the dish. Using OCT imaging, we imaged the mid-sagittal plane of the brain and ventricles. When all five flow fields were visible in the same frame, we recorded the 2D and 3D images. We then imaged the dorsal cardinal vein to document venous blood flow.

We then flipped the dish over and removed the coverslip to expose the heart. Using borosilicate fine tip needles, we dissected the heart out. We replaced the coverslip, turned the dish right side up, then overlaid the agarose with 1/9× MR with gentamycin and let the embryo recover for 25–30 min. After the recovery period, we imaged the dorsal cardinal vein to verify no blood flow and then reimaged the ventricles.

### Heart ablation at stage 40

Wild type tadpoles were raised to stage 40- the stage when their hearts and outflow tracts were visible under a stereomicroscope. The embryos were embedded in 1% low melt agarose in 1/9× MR and positioned laterally to access cardiac sacs. Using fine tip needles outflow tracts were dissected out, then tadpoles were gently removed and transferred to fresh 1/9× MR with 0.1% gentamycin. They, along with stage-matched unmanipulated control embryos were then incubated at 25 °C until they reached stage 46 when they were first imaged using OCT to confirm lack of cardiac circulation and then fixed and prepared for in situ hybridization as described below.

### Intraventricular microbead injections

0.81 µm or 4.61 µm diameter polystyrene microsphere suspensions (Bangs Laboratories) were diluted in artificial CSF (124 mM NaCl, 2 mM KCl, 1.6 mM MgSO_4_ -7H_2_O, 1.3 mM KH_2_PO_4_, 24 mM NaHCO_3_, 2 mM CaCl_2_-2H_2_O, 22 mM d-( +)-Glucose) from the original suspensions to a final concentration of 0.25% solids (0.81 µm beads) or 0.5% solids (4.61 µm beads). After embedding stage 30, and 49 tadpoles in 1.5% low melt agarose, they were injected with the bead suspension into the ventricular space at the hindbrain (stage 30) or into the third ventricle (stage 47, 48, and 49) using the microinjection apparatus. Injection volumes were 4 nL for stage 30 tadpoles and 25 nL for stage 47, 48, and 49 tadpoles.

### Chemical CSF flow abatement

Separate injection solutions were prepared in artificial CSF of nickel(II) chloride hexahydrate (50 mM) (Sigma Aldrich) and sodium orthovanadate (10 mM) (Sigma Aldrich). After the injection of beads into the third ventricle of agarose embedded stage 49 tadpoles and recording pre-treatment OCT movies, we injected 25 nL of one of the above solutions into the same location as the beads injection, then recorded OCT movies at 5 min time intervals.

### Immunohistochemistry

For *Xenopus tropicalis,* stage 46 tadpoles were fixed in 4% paraformaldehyde in PBS for 2 h at room temperature. The tadpoles were washed 3 × 15 min with PBS, and then the brains were dissected out. The brains were incubated for 1 h at room temperature in blocking buffer (3% BSA, 0.2% Triton X-100 in PBS), then incubated overnight at 4 °C in primary antibody against glutamylated tubulin (GT335, AdipoGen) diluted 1:1000 in blocking buffer [48]. Samples were then washed 3 × 15 min with PBS then incubated for 2 h at room temperature in secondary antibody (anti-mouse Alexa Fluor 488 or Alexa Fluor 594, Invitrogen) diluted 1:500 in blocking buffer, followed by 3 × 15 min washes with PBS. The brains were incubated for 1 h at room temperature in Alexa Fluor 647 phalloidin (Invitrogen) diluted 1:100 in blocking buffer, washed 3 × 15 min with PBS, then mounted between coverslips in ProLong Gold antifade reagent (Invitrogen). Immunostained brains were imaged on a Zeiss 710 confocal microscope.

For *Xenopus laevis*, stage 46 tadpoles were stained whole-mount according to Willsey et al. 2018 [49] using the primary antibody against glutamylated tubulin (GT335, AdipoGen) diluted 1:100. Following staining, brains were dissected and mounted in Vectashield (Vector Laboratories) and imaged on a Leica SP8 confocal microscope.

### In situ hybridization

Wildtype tadpoles were imaged using OCT at stage 46 to confirm typical ventricle size, shape, and CSF flow, then fixed with MEMFA (100 mM MOPS (pH 7.4), 2 mM EGTA, 1 mM MgSO_4_, 3.7% (v/v) formaldehyde) for 2 h at room temperature. The tadpoles were then washed multiple times with 100% ethanol, then stored at − 20 °C. *c21orf59* morphants were imaged using OCT at stage 46 and tadpoles with aqueductal stenosis were recorded and selected to be fixed as above with MEMFA.

In situ hybridization was performed according to the standard protocols [41]. Here we used ethanol for all dehydration steps, and 4% paraformaldehyde + 0.1% glutaraldehyde in PBS was used as the post staining fixative for 20 min at room temperature instead of Bouin’s fixative.

#### Antisense probes

**Table:** 

Gene	NCBI reference	Linearization restriction enzyme	Probe making polymerase
*lhx1*	NM_001100228.1	ClaI	T7
*emx1*	NM_001005459.1	ClaI	T7
*en2*	XM_002932479.4	SpeI	T7

### Quantification and statistical analysis

Data was analyzed using Prism7 statistical software. For comparison between pre and post cardiac ablation we utilized the Wilcoxon matched-pairs (non-parametric, paired) and used before-after graph including standard error of the mean. Significance was determined when the p value is lower than 0.01. For comparison between controls and *c21orf59* morphants we utilized Mann–Whitney test (nonparametric, unpaired) and used violin plot graph to show the distribution of the data. Significance was determined when the p value is lower than 0.01.

## Results

### OCT imaging in *Xenopus* enables in vivo temporal examination of ventriculogenesis as well as CSF flow initiation and maturation without manipulation

**Fig. 1:**
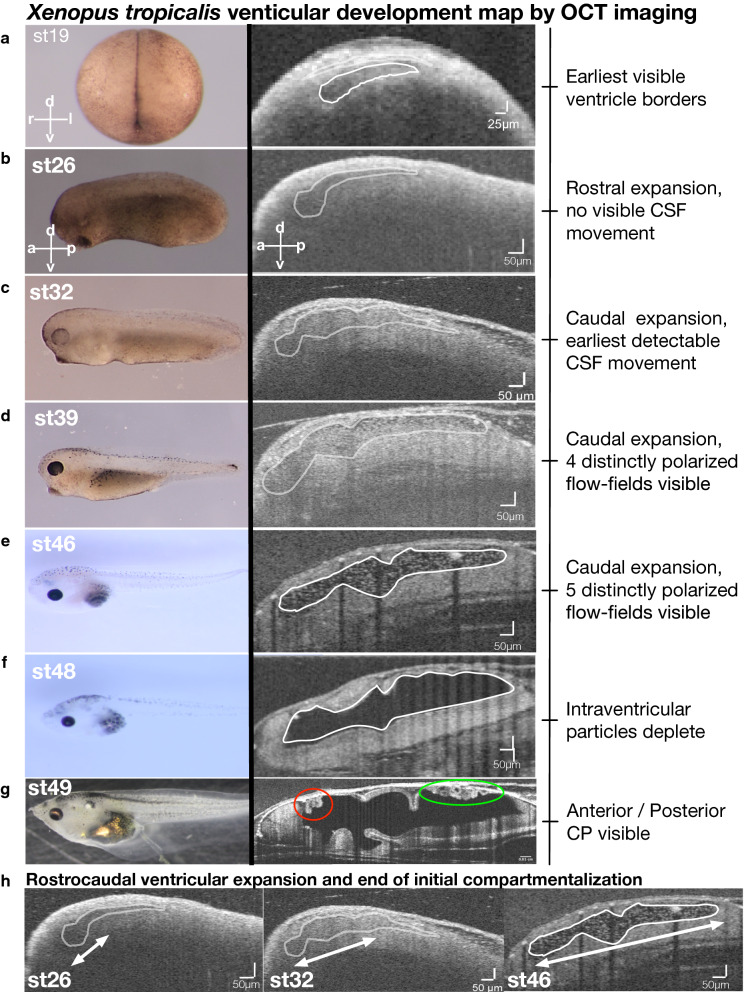
*Xenopus tropicalis* ventricular development map by OCT imaging. Widefield and mid-sagittal plane in vivo OCT imaging of an individual embryo at various stages to track ventricular development. **a** Stage 19; the earliest stage at which ventricular space is visible. **b** Stage 26; rostral expansion of the ventricular space is shown. **c** Stage 32; caudal expansion and the earliest detectable intraventricular particle movement (Additional file 2: Movie S1). **d** Stage 39; continued caudal expansion and 4 distinct polarized flow fields are visible (Additional file 2: Movie S1). **e** Stage 46; further caudal expansion and 5 distinct polarized flow fields are visible (Additional file 2: Movie S1). **f** Stage 48; intraventricular particle density diminishes. **g** Stage 49; Anterior (red circle) and posterior (green circle) choroid plexus visible. **h** Rostrocaudal ventricular expansion progression. CSF: cerebrospinal fluid; CP: choroid plexus; OCT: optical coherence tomography, a: anterior; p: posterior; d: dorsal; v: ventral

We have previously shown that using a *Xenopus* Optical Coherence Tomography platform, in a non-destructive fashion, we can detect global CSF flow mapped to CNS structures in the *Xenopus* tadpole [34]. To address the question of what determines the *Xenopus* embryonic CSF circulation, we again capitalized on the power of OCT imaging. Despite the optically dense yolk, unexpectedly, OCT imaging allowed us to serially examine each developmental stage of the *Xenopus* embryo, starting from stage 19 (post-neurulation, ~ 16 h post-fertilization at 25 °C) until stage 49 (tadpole) (Nieuwkoop—Faber staging. ~ post-fertilization day 4 at 25 °C) (Fig. 1, Additional file 1: Fig. S1). Immediately around neural tube closure at stage 18–19, we observed the first visible ventricular structure by OCT imaging as a slit-like space at the rostral–dorsal side of the embryo (Fig. 1a). This slit-like space initially expanded rostrally, forming an enlarged, bulbous area visible at stage 26 (Fig. 1b), which then expanded caudally and eventually formed compartments connected by aqueducts (Fig. 1c–h, Additional file 1: Fig. S1). At stage 49, anterior and posterior choroid plexuses become visible (Fig. 1g—red and green circle).

**Fig. 2:**
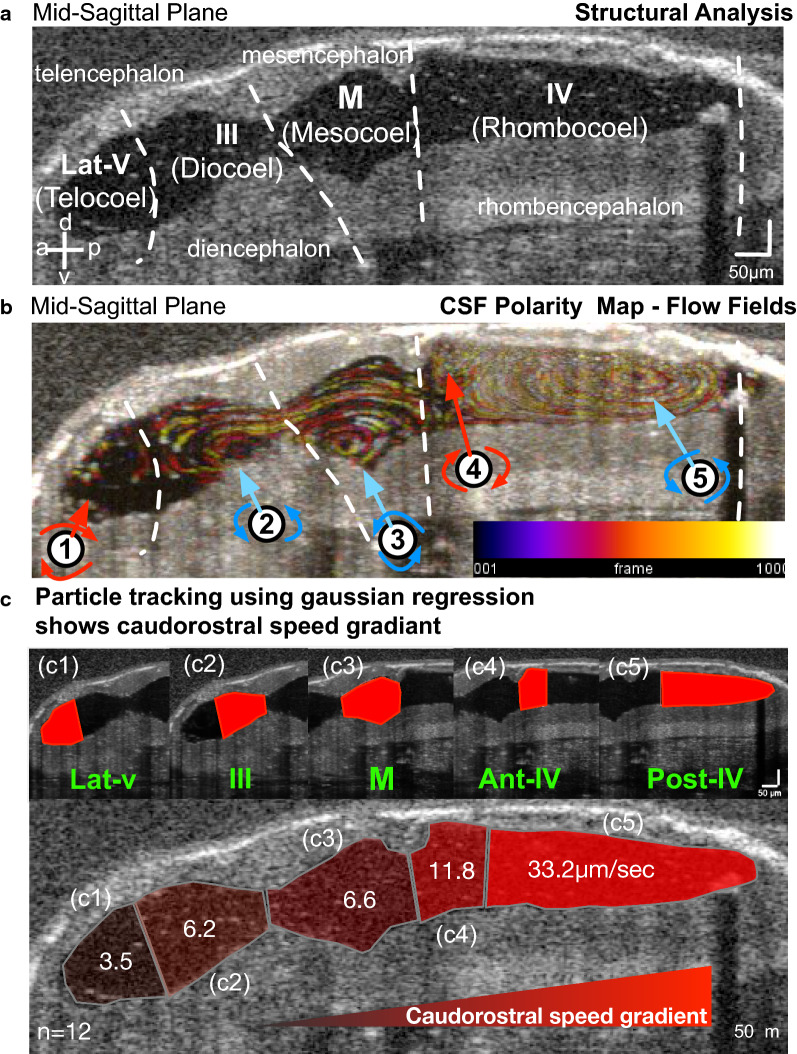
Particle tracking with Gaussian process regression enables compartmental CSF flow speed measurements. **a** Mid-sagittal plane in vivo OCT imaging of a stage 46 tadpole outlining brain structures and ventricular spaces. **b** CSF polarity map based on temporally color-coded frames 1–1000 at the mid-sagittal plane delineates particle trajectories of 5 discrete flow fields (labeled 1–5). FF1: telencephalic, FF2: diencephalic, FF3: mesencephalic, FF4: anterior rhombencephalic, FF5: posterior rhombencephalic (red: clockwise, blue: counterclockwise). **c** Compartmentally-matched median CSF flow speed based on particle tracking using Gaussian process regression showing a caudo-rostral speed gradient. c1) Lateral ventricle: 3.5 µm/s, c2) III ventricle: 6.2 µm/s, c3) Midbrain ventricle: 6.6 µm/s, c4) Anterior IV ventricle: 11.8 µm/s, c5) Posterior IV ventricle: 33.2 µm/s. CSF: cerebrospinal fluid; a: anterior, p: posterior, d: dorsal, v: ventral, Lat-v: lateral ventricle, III: 3rd ventricle, M: midbrain ventricle, IV: 4th ventricle

Simultaneous to the structural examination, we also analyzed the CSF flow without manipulation of the intraventricular space. This analysis relies on the presence of the endogenous particles within the fluid-filled cavities of the ventricles [34]. OCT imaging can resolve these particles, and using particle tracking techniques; we mapped the CSF flow pattern as we previously explained [34]. As the ventricles enlarged, by stage 32, we were able to see the first distinguishable movement of the particles inside the ventricle without a polarized flow pattern. We marked this time as the first visible pre-choroidal CSF movement by OCT imaging (Fig. 1C, Additional file 2: Movie S1). As the caudal expansion continued, eventually, 4 flow fields became visible at stage 39 (Fig. 1d, Additional file 2: Movie S1) and 5 flow fields at stage 46 (Fig. 1e). At stage 46, intraventricular particles became sparser and were few by stage 48 (Fig. 1f) and absent by stage 49 (Fig. 1g). We have previously named these five discretely polarized flow fields (FF) nested within four ventricles as FF1: Telencephalic flow, FF2: Diencephalic flow, FF3: Mesencephalic flow, FF4: Anterior rhombencephalic flow, FF5: Posterior rhombencephalic flow (Fig. 2a, b) [34].

To discretely examine and quantify changes in individual compartmental flow velocities, we developed and published a method to calculate dense velocity estimates based on particle tracking (see methods for details [44]). Using our Gaussian post-processing, here we showed the median speed for each ventricular compartment (Fig. 2c). We defined the compartments' borders based on the anatomical borders of the telencephalon, diencephalon, mesencephalon, and rhombencephalon, respectively. The results demonstrated the highest flow speed at the most caudal portion of the 4th ventricle (33.2 µm/s) and decreased speed at the anterior portion of the 4th ventricle (11.2 µm/s). The CSF flow speed continued to decrease in mesencephalic ventricle (6.6 µm/s), 3rd ventricle (6.2 µm/s), and the slowest flow was measured in the most rostral lateral ventricle (3.5 µm/s) (n = 12) (Fig. 2c). Together, we built a flow speed map based on the magnitude of the velocity that showed a decreasing flow rate from caudal to rostral. The caudal CSF flow was ~ tenfold higher than the rostral flow, and we can now use these compartmental CSF flow speeds for comparison studies.

**Fig. 3:**
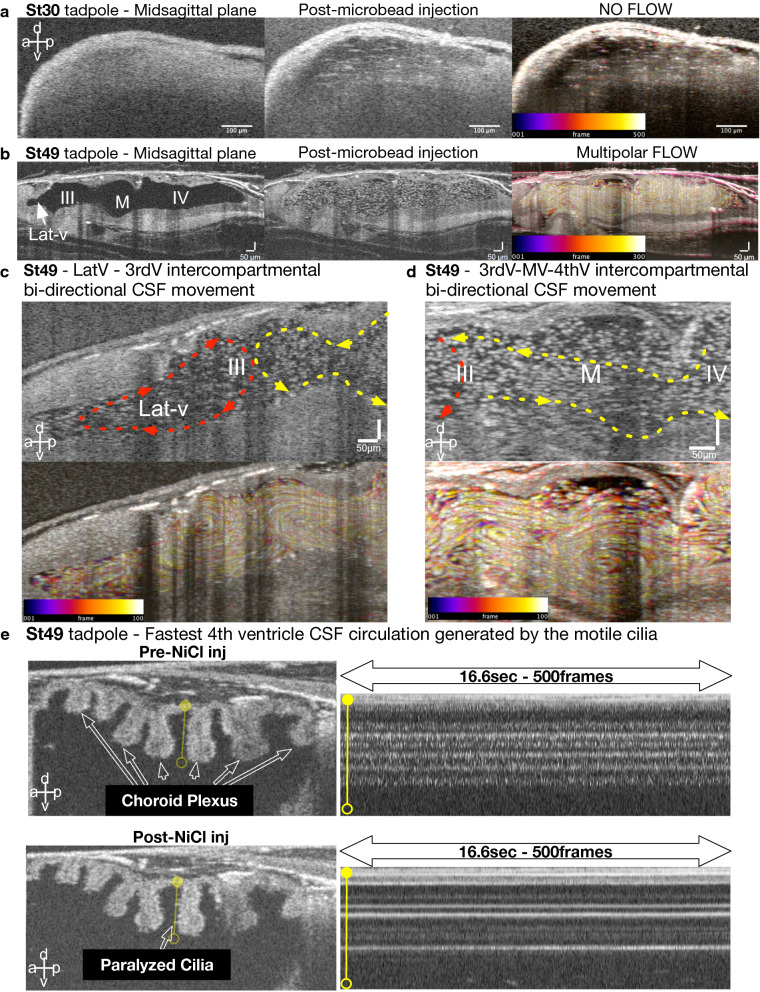
Late pre-metamorphosis tadpole displays complex compartmental CSF circulation and bidirectional intercompartmental mixing. **a** Mid-sagittal plane in vivo OCT imaging of stage 30 tadpole before and after microbead injection. Temporal color-coded image shows suspended, static bead (Additional file 3: Movie S2). **b** Mid-sagittal plane in vivo OCT imaging of stage 49 tadpole before and after microbead injection. The temporal color-coded image shows multiplanar CSF circulation (Additional file 4: Movie S3). **c** Focused image shows the aqueduct between the lateral ventricle and 3rd ventricle. The trajectory of the CSF circulation is shown by the red and yellow arrows (Additional file 5: Movie S4). **d** Focused image shows the aqueducts between the 3rd, midbrain (MV) and 4th ventricles (IV). The trajectory of the CSF circulation is shown by the red and yellow arrows (Additional file 6: Movie S5). (E) Focused 4th ventricle OCT image shows the choroid plexus projections (white arrows). Post-NiCl intraventricular injection, OCT image allows visualization of the static ependymal cilia (white arrow—Additional file 6: Movie S6). Along the yellow line, the kymograph showing motile cilia’s beating along the choroid plexus surface, which stops post NiCl injection

In summary, OCT imaging enabled continuous visualization of *Xenopus* embryonic ventricular development from as early as post neurulation. The ventricular space enlarges in a rostrocaudal fashion throughout the early tailbud stages (Stage 22–28). Then, five flow fields emerge, circulating and mixing CSF between compartments throughout the late tailbud stages, where we show a caudal to ostral decreasing CSF flow rate (Stage 30–44). We further expanded the interrogation of the earlier (before stage 32) and later stages (after stage 46) by introducing intraventricular polystyrene beads into the ventricular space (Fig. 3). At stage 30, beads were clearly visible and remained static (Fig. 3a, Additional file 3: Movie S2), further confirming the lack of CSF movement. We concluded that there is no CSF movement detectable by OCT imaging (Additional files 2, 3: Movie S1, S2) before stage 32, at the early tailbud stages, when the ventricles are expanding.

### Late pre-metamorphosis tadpole displays complex compartmental CSF circulation and bidirectional intercompartmental mixing

When we evaluated the late stage 49 tadpole (limb buds form-metamorphosis begins) with intraventricular bead injection, we noted an intricate CSF circulatory pattern (Fig. 3B, Additional file 4: Movie S3) where CSF flow is not only polarized within each ventricular compartment but also across the aqueducts. We noted bidirectional flow across all the aqueducts where the dorsal flow is polarized towards the rostrum and ventral flow to the caudal region (Fig. 3C, D, Additional files 5, 6: Movie S4, S5), establishing a continuous intercompartmental mixing. At this stage, 3rd and 4th ventricle choroid plexuses are visible (Additional file 1: Fig. S1B), and 4th ventricle choroidal region continues to generate the fastest CSF movement. Using OCT imaging, we were able to visualize ciliary motion along the 4th ventricle choroidal surface (Fig. 3e—Additional file 7: Movie S6); when we introduced NiCl [50–52] to ablate ciliary movement we were able to visualize the cessation of the ciliary beating (Fig. 3e—Additional file 7: Movie S6) and the lack of CSF movement (Additional file 8: Movie S7), confirming that, indeed, fastest CSF flow in the 4th ventricle is generated by the ependymal cilia. We did not detect any pulsatile flow from the choroid plexus. Next, we set out to qualitatively examine the potential for cardiac pulsations to contribute to CSF flow.

### CSF circulation forms independent from cardiac forces in the *Xenopus* embryonic brain

**Fig. 4:**
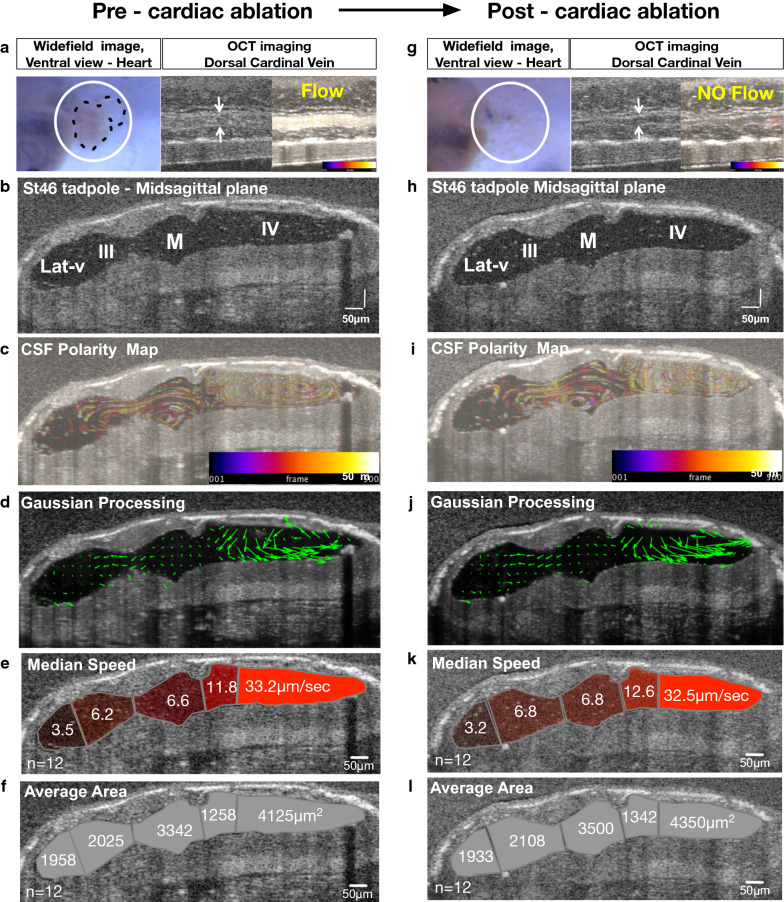
In *Xenopus tropicalis* polarized embryonic CSF circulation forms independent of cardiac forces. **a**, **g** Widefield image of the heart vicinity and OCT image of the dorsal cardinal vein of a stage 46 tadpole. White circle outlines the cardiac sac. Black dotted line marks the heart and the outflow tract of the tadpole pre-cardiac ablation. The absence of the heart is shown post-cardiac ablation. White arrows point to the outer vein walls and a temporally color-coded image indicates the presence or absence of blood flow. **b**, **h** Mid-sagittal plane in vivo OCT image of the ventricular space. **c**, **i** CSF polarity flow map. **d**, **j** Post-Gaussian processing CSF flow map. **e**, **k** Median compartmental median CSF flow speed. **f**, **l** Average compartmental area. (Additional file 10: Movie S8). CSF: cerebrospinal fluid; OCT: optical coherence tomography, Lat-v: lateral ventricle, III: 3rd ventricle, M: midbrain ventricle, IV: 4th ventricle

The *Xenopus* cardiovascular system develops within 72 h and remains optically accessible throughout the early stages of development [53]. Most importantly, oxygen delivery in tadpoles relies on simple diffusion through the skin rather than on cardiac output [54]. Even in the presence of a non-beating heart, tadpoles survive for several days; therefore, *Xenopus* is ideal for analyzing CSF dynamics in tadpoles without cardiac circulation [54, 55]. To test the contribution of the cardiac forces to the CSF circulation, we imaged the stage 46 tadpole ventricular system pre and post cardiac ablation (Fig. 4). First, we imaged the ventricular space as well as the cardinal vein to document normal venous flow, ventricular structures, polarized flow fields, flow speed, and finally ventricular size (Fig. 4a–f). Then we accessed the cardiac sac and removed the heart by microdissection. The cessation of the cardiac circulation was confirmed by the loss of cardinal vein flow (Fig. 4g), and we reimaged the ventricular space for CSF flow speed, polarity, and ventricular size (Fig. 4h–l). We did not detect any changes to CSF polarity or speed, nor did we find any difference in the size, shape and volume of the ventricular space (Figs. 4, Additional files 9, 10: Fig. S3, Movie S8).

**Fig. 5:**
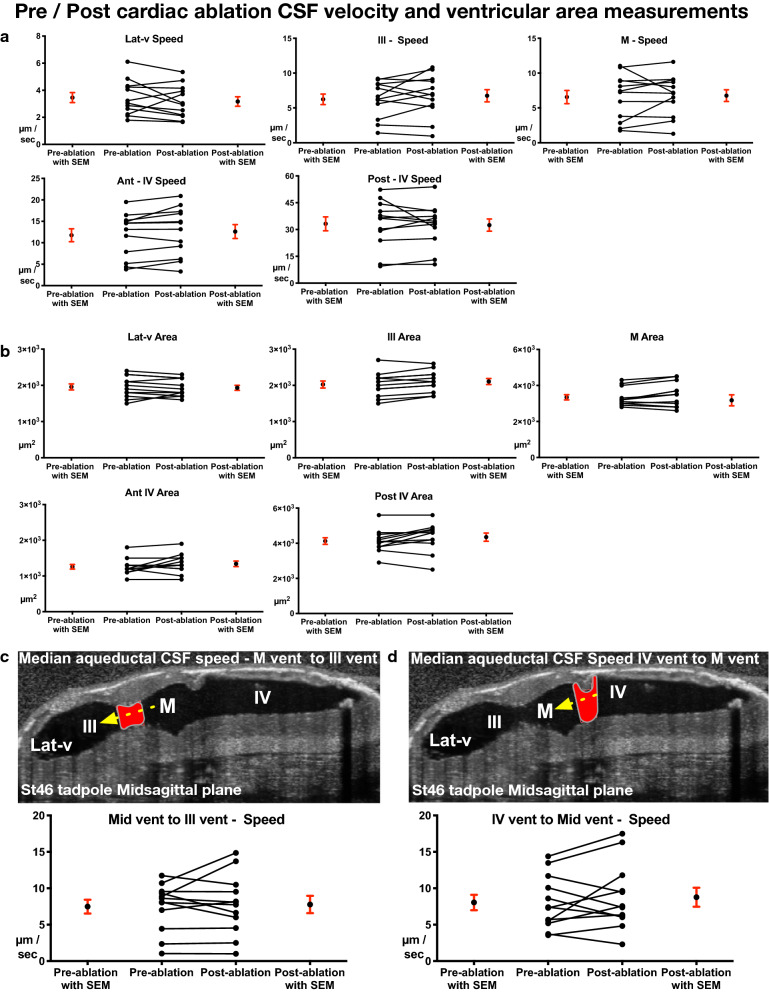
Pre/post cardiac ablation CSF flow speed and ventricular area measurements (Stage 46). **a** Median compartmental CSF flow speed of the Lat-V, 3rd ventricle, midbrain ventricle, anterior 4th and posterior 4th ventricle presented with before-after graphs. **b** Average compartmental cross-sectional area of the Lat-V, 3rd ventricle, midbrain ventricle, anterior 4th and posterior 4th ventricle presented with before-after graphs. **c** Median compartmental flow speed along the aqueducts between the 3rd and midbrain ventricles, and the 4th and midbrain ventricles shown with before-after graphs. Red area outlines the cross section of where the particle velocimetry is applied, yellow-dotted arrow indicates the direction of flow. Lat-v: lateral ventricle, III: 3rd ventricle, M: midbrain ventricle, IV: 4th ventricle

We further focused on the interventricular spaces where narrow aqueducts exist and CSF mixes between compartments. We specifically quantified the velocities across the aqueducts connecting the 3rd ventricle to the midbrain ventricle and the midbrain ventricle to the 4th ventricle, in an attempt to detect subtle differences in CSF flow speed since at these connections the CSF flow is slower relative to the intraventricular flow, as previously reported in zebrafish model. Nevertheless, we did not appreciate a difference (Fig. 5c), concluding that both compartmental and global CSF circulation, as well as interventricular mixing, is independent of the embryonic cardiac forces in *Xenopus*.

Not only did we not detect involvement of cardiac forces up to tailbud embryonic CSF circulation, but we also did not detect any pulsatile flow up to stage 46 tadpole. We next interrogated the late tailbud pre-metamorphoses stages. At stage 49, we first injected microbeads to track CSF circulation, followed by the nickel(II) chloride hexahydrate or sodium orthovanadate [56, 57] injection to slow the ciliary beating. We detected a weak pulsatile flow around the hypothalamic region only (Additional file 11: Movie S9). Even at these late stages, pulsatile flow remained undetectable in the primary ventricular system in *Xenopus. *With multiple washes, we were able to recover the cilia-driven flow (Additional files 12, 13: Movie S10, S11).

### Cilia driven embryonic CSF circulation is required for rostral CNS development

**Fig. 6:**
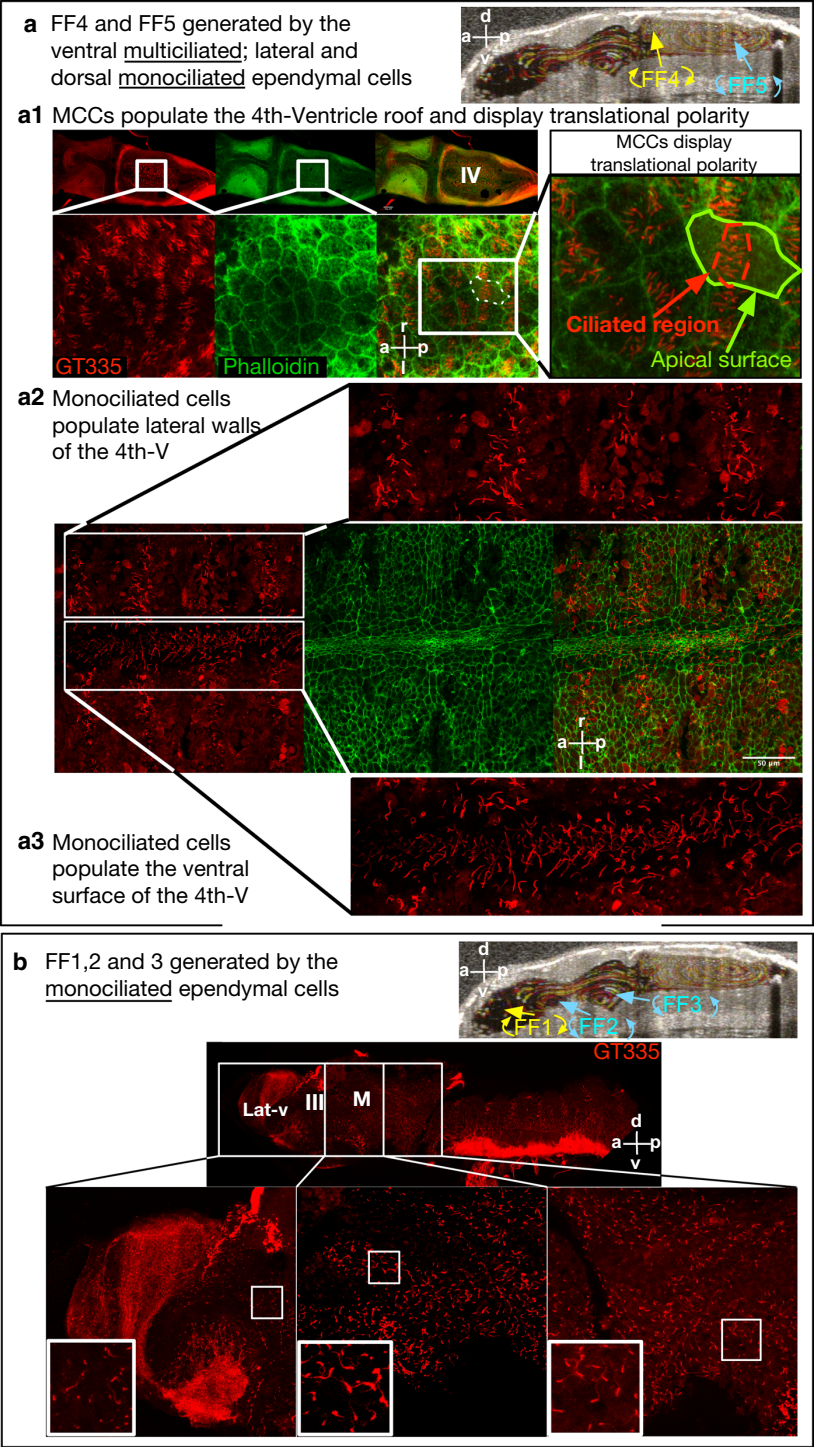
*Xenopus tropicalis* ventricular cilia distribution in brain explants (Stage 46). **a** Mid-sagittal OCT image showing flow fields 4 and 5. **a1** Brain stained with an anti-GT335 antibody (red) which labels cilia, and with phalloidin (green) which labels actin to mark cell borders. The dorsal fourth ventricle roof is populated with MCCs that display translational polarity. **a2** The lateral walls of the 4th ventricle displays monociliated cells. **a3** The ventral surface of the 4th ventricle populated with monociliated cells. **b** The lateral, third, and midbrain ventricles show a dense population of monociliated cells. Lat-v: lateral ventricle, III: 3rd ventricle, M: midbrain ventricle, IV: 4th ventricle, CA: cerebral aqueduct, FF: flow field

In adult brains, besides the cardiac forces, the second known driver of the embryonic CSF circulation is the ciliated ependymal cells. We and others reported that when ependymal cilia are genetically depleted, CSF circulation and proper neurodevelopment is altered [21, 34]. With the knowledge that cardiac forces do not contribute to *Xenopus* embryonic CSF circulation, we first examined the distribution of the cilia along the ventricular surface and the impact of cilia-driven flow on CNS development. Previous reports have described that frog ventricles are densely populated with ciliated cells [58–60]. Here we focused on the regions where we delineated five distinct flow fields. When we examined the rhombencephalic dorsal surface in both *Xenopus* tropicalis and laevis species, where 4th and 5th flow fields generate the fastest CSF circulation, we observed translationally polarized multiciliated ependymal cells, where cilia are confined to the center of the apical surface (Fig. 6A1, Additional file 14: Fig. S2). When we focused on the lateral wall (Fig. 6A2), rather than multiciliated cells, we observed monociliated cells with discrete long cilia. Similarly, the ventral surface of the 4th ventricle is populated with monociliated cells (Fig. 6A3). In comparison, the more rostral area along with the midbrain, 3rd, and lateral ventricular surfaces showed a dense network of monociliated cells expressing long individual cilia (Fig. 6B) and a limited multiciliated cell distribution near the pineal gland and the anterior choroid plexus region (Additional file 14: Fig. S2).

**Fig. 7:**
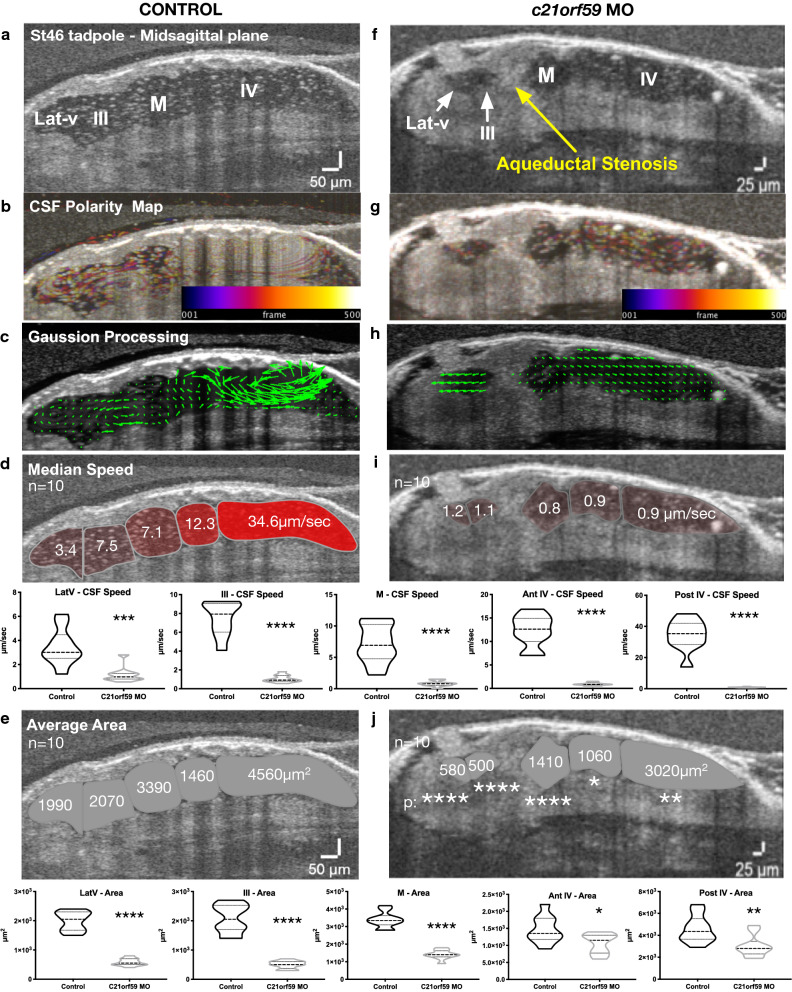
Ependymal cilia driven flow is most impactful on rostral development. Mid-sagittal plane in vivo OCT imaging of stage 46 control and *c21orf59* morphant tadpoles. **a**, **f** Midsagittal ventricular space, yellow arrow points the aqueductal stenosis. **b**, **g** CSF polarity flow map. **c**, **h** Post-Gaussian processing flow map. **d**, **i** Median compartmental flow speed. **e**, **j** Average compartmental area. *p < 0.01, **p < 0.001, ***p < 0.001, ****p < 0.0001

To analyze the impact of cilia-driven flow on CNS development in *Xenopus*, we used an established morpholino oligo (antisense oligonucleotide) to knockdown *c21orf59* (cilia and flagella associated protein 298). *c21of59* is required for ciliary dynein arm assembly, which is known to regulate motile cilia function [42]. When *c21orf59* is lost, motile cilia morphologically form correctly but are unable to beat, remaining paralyzed. We had previously shown that when we knocked down *c21orf59* (Fig. 7), we observed two significant outcomes: 1) as expected, CSF circulation was globally lost, and 2) tadpoles developed aqueductal stenosis, a hallmark of obstructive hydrocephalus in the setting of abnormal rostral development [34]. Interestingly, when we further analyzed the ventricle size and shape here, there was a striking difference in the rostral development when compared to the caudal development of the brain (Fig. 7e). When we globally measured the ventricular volume, it was smaller in morphants (Additional file 9: Fig. S3). When we measured the ventricle area of the morphants at the mid-sagittal plane, the lateral ventricle (580 µm^2^) and 3rd ventricle (500 µm^2^) were much smaller than controls (1990 and 2070 µm^2^, respectively) whereas the midbrain ventricle area (1410 µm^2^), anterior 4th (1060 µm^2^) and posterior 4th ventricles (3020 µm^2^) were much closer to control (Fig. 7e, j). Thus we conclude that embryonic CSF circulation solely relies on the motile ciliated ependymal cells, lacks cardiac driven pulsatile flow and the loss of this cilia-driven flow affects rostral ventricle and brain development more dramatically when compared to the caudal regions.

### Cilia driven CSF circulation conveys neuroprogenitor spatial organization in embryonic forebrain development, cardiac forces have no effect

**Fig. 8:**
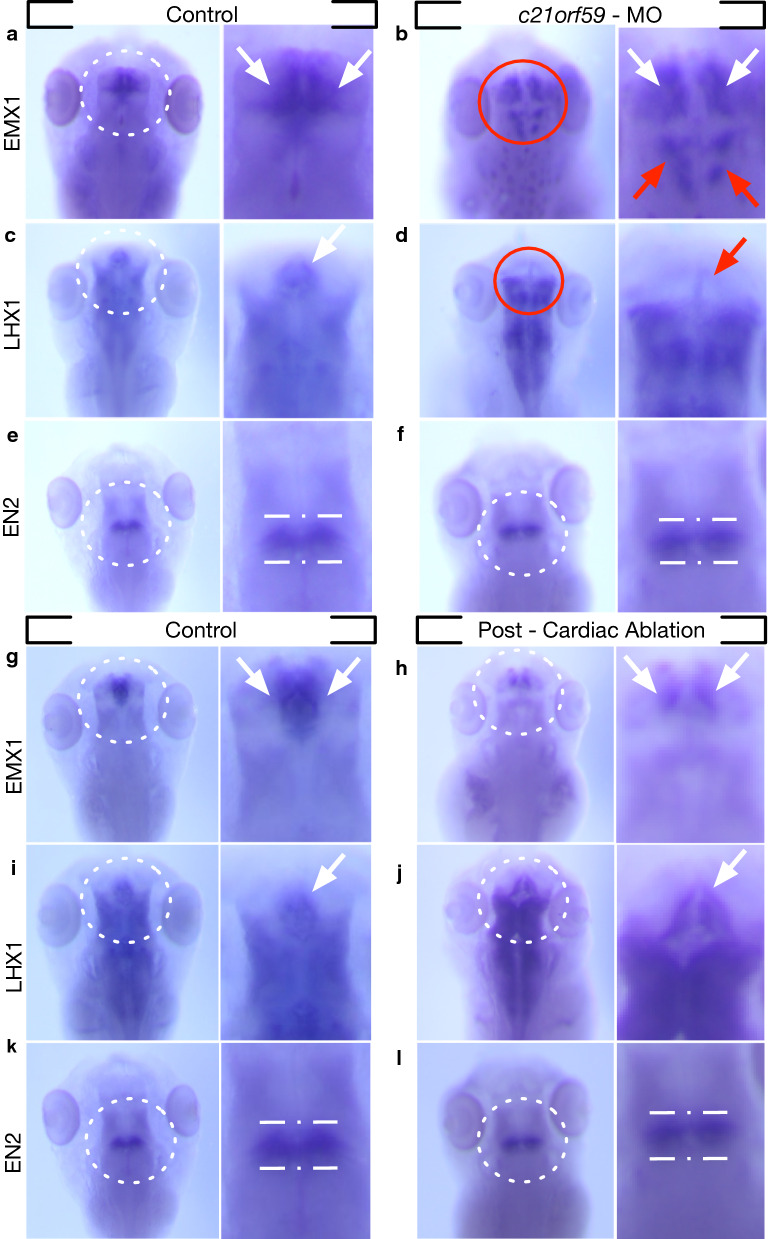
Ependymal cilia driven flow is most impactful on rostral development and cardiac forces have no effect. **a** Normal expression of *emx1* mRNA in a stage 46 wild-type tadpole. Expression is confined to the dorsal telencephalic area. White arrows in the magnified region mark the dorsal telencephalic region where *emx1* is strongly expressed. **b** *c21orf59* knockdown results in expanded *emx1* expression caudally to the diencephalon and mesencephalon regions indicated by red arrows. **c** Normal expression of *lhx1* mRNA in a stage 46 wild-type tadpole. Expression is strong around the thalamic region as indicated by a white arrow and extends to the diencephalon. **d** *c21orf59* knockdown results in a loss of the *lhx1* expression in the telencephalon, indicated by a red arrow. **e** Normal expression of *en2* mRNA in a stage 46 tadpole. Expression localizes to the midbrain-hindbrain boundary outlined with dotted white line. **f** *c21orf59* knockdown results in no observed change in the *en2* expression pattern. **g**, **i**, **k** Normal expression of *emx1*, *lhx1*, and *en2* mRNA when compared with **h**, **j**, **l** heartless tadpoles shows no observed changes in expression patterns

The marked rostral morphological changes in the setting of loss of CSF flow prompted us to examine telencephalic neuroprogenitor cells to understand better the nature of this dysregulation. We examined by in situ hybridization three spatiotemporally regulated transcription factors; *emx1, lhx1*, and *en2: emx1* (empty spiracles homeobox 1) is known to regulate dorsal telencephalon development [61]. The expression of *emx1* in humans, mice, and *Xenopus* is spatially restricted to the cerebral cortex [62, 63]. As shown in Fig. 8a (white arrows), we also observed this isolated dorsal telencephalic expression pattern in unmanipulated tadpoles. When we knocked down *c21orf59* with morpholino and confirmed the paralysis of motile cilia by the loss of CSF circulation by OCT imaging, we observed that *emx1* dorsal telencephalic expression was mis-patterned in these tadpoles. Expression of *emx1* was not lost or diminished but instead, was no longer limited to the dorsal telencephalon. In morphants, we were able to see ectopically-expressed *emx1* within the diencephalon, extending towards the mesencephalon (Fig. 8b—red arrows, Additional file 15: Fig. S4). This rostrocaudal patterning defect raised the question of whether this dysregulation is valid for the known secondary organizers.

We know that along the rostrocaudal axis, thalamus—prethalamus (*zli*—*zona limitans intrathalamica*) and the midbrain-hindbrain (isthmic organizer) transition zones harbor regions that act as secondary organizers that regulate patterning following neural tube formation [64–67]. The *zli* is an evolutionarily conserved structure that demarcates the boundary between pre-thalamus and thalamus (posterior diencephalon – anterior diencephalon) and is referred as the mid-diencephalic organizer [67] where Sonic hedgehog (Shh) is secreted to regulate thalamic development. We asked whether these secondary organizers would be affected by the loss of CSF circulation. To examine the *zli*, we investigated LIM homeobox 1 (*lhx1*), a transcription factor known to regulate differentiation and morphogenetic tissue movement in head development [68–71]. In both mice and *Xenopus, lhx1* depletion leads to loss of anterior head structures [68, 72] and is known to be expressed in the *zli.* As shown in Fig. 7C (white arrow), *lhx1* marks the thalamic region in *Xenopus* stage 46 tadpole as well as the midbrain region. This pattern was explicitly disrupted at the telencephalic region and was relatively conserved elsewhere when the cilia were paralyzed, and the CSF circulation ceased in *c21orf59* morphants (Fig. 8D – Additional file 15: Fig. S4).

We then examined a more caudal region, the midbrain-hindbrain boundary using the *en2* marker. The *engrailed gene* is a subset of highly conserved homeobox genes that are expressed at the midbrain-hindbrain junction (Fig. 8e) [73]. Specifically in *Xenopus, en2* is one of the earliest markers of neural plate regionalization [73]. Interestingly when we impaired the CSF circulation, we did not observe a change in *en2* expression. The borders were well delineated, and expression levels were grossly similar to unmanipulated tadpoles (Fig. 8F, Additional file 15: Fig. S4).

Finally, we asked whether cardiac ablation would affect neuroprogenitor patterning. At stage 40, we dissected hearts and raised tadpoles two days to stage 46 when we confirmed the presence of normal CSF circulation and lack of cardiac circulation. As expected, these tadpoles showed global swelling in the setting of no cardiac function leading to renal failure, yet we observed no difference in the expression of *emx1, lhx1*, or *en2* (Fig. 8 G-L, Additional file 15: Fig. S4).

## Discussion

**Fig. 9:**
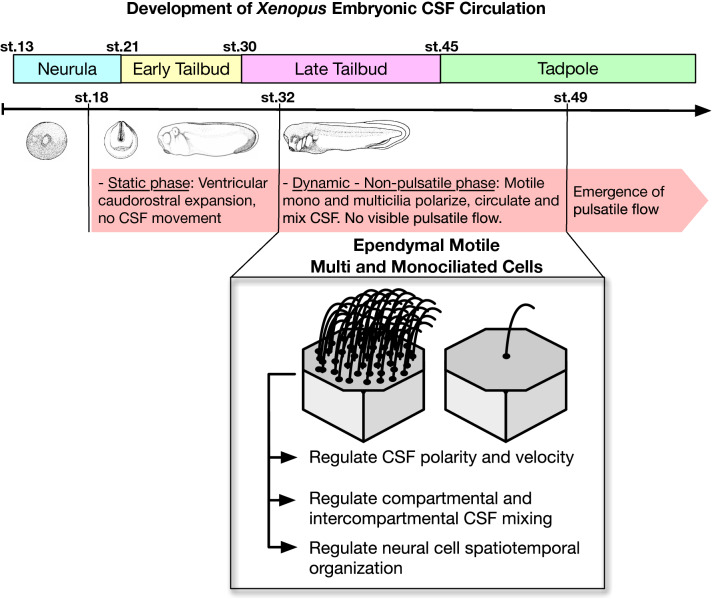
Development of Xenopus embryonic CSF circulation

We have shown that vertebrate *Xenopus* ventriculogenesis and CSF circulation development can be tracked in the embryonic brain in real-time without manipulating the ventricular space. The *Xenopus* embryo can survive until at least feeding tadpole stages (post-fertilization day 5) without a heart [54, 55]. Exploiting these unique features, we have constructed the first in vivo OCT map of the *Xenopus* developing central nervous and ventricular systems during the genesis and maturation of the embryonic CSF circulation. This examination has uncovered multiple novel observations in the *Xenopus* embryonic nervous system (Fig. 9).

Immediately after the neural tube closure, we were able to visualize in vivo the initial rostral ventricular expansion up until the late tailbud stages, when we were able to observe the first premature CSF movement at the caudal aspect of the ventricle. There are limitations to the native CSF flow assessment: (1) We analyze the CSF movement using particle tracking, which relies on the endogenous particles that are readily present, suspended within the ventricular space. Therefore, the unmanipulated, endogenous CSF movement`s detection is limited by the OCT resolution and the availability of these particles. The earliest time point where we were able to detect CSF movement by OCT imaging was at stage 32—tailbud stage. (2) As the density of the intraventricular particles diminishes over time, particle tracking improves. Currently, available particle detection algorithms are more sensitive and able to detect and track more efficiently when particle density is low enough to discriminate a particle from the background. We can quickly qualitatively assess CSF movement at the earliest stages of CSF circulation by following these particles. However, we cannot precisely outline polarity or reliably quantify particle speeds at the very early stages. As OCT imaging technology advances, we anticipate an improvement in particle tracking when the density is high.

We showed that during the rostrocaudal ventricular expansion, CSF circulation is compartmentalized in a retrograde caudal-rostral direction. Circulation initiates caudally and eventually forms five mature flow fields, and this can be easily tracked in *Xenopus*. When we quantified the median CSF speed, interestingly, we observed that the speed also is markedly different along the caudal-rostral axis. The presence of such a difference in the magnitude of ~ tenfold between the forebrain and hindbrain hints at potential compartmental differences in the CSF circulation's regulatory effects. When we couple the different ependymal cilia populations with our CSF polarity map, we see that the discrete motile mono and multicilia polarize circulation even within the same compartment. As we know, near-wall CSF dynamics do regulate neuroprogenitor migration, proliferation, and axon pathfinding [12], yet the mechanism remains to be elucidated. Based on our observation, the differences in CSF circulatory speed and cilia type may add another layer of questions, such as the degree of velocity and different types of ciliopathies that may have different implications. Further studies are required to manipulate the flow polarity and speed to understand better the implications of this finely tuned CSF flow gradient on neurogenesis.

New animal models, especially zebrafish, paired with in vivo imaging, revealed much about the embryonic CSF circulation. Fame et al. showed that CSF's directional movement in the zebrafish embryo, which preferentially moved from the diencephalic to the rhombencephalic ventricle, reversed between the early and late larval stages, and one of the key results was that CSF flow was partially dependent on the heartbeat [20]. In *tnnt2a-/-* mutants (that lack a heartbeat), maximal CSF flow (V_max_) was lower from the diencephalic and mesencephalic ventricles to the rhombencephalic ventricle. The authors concluded that the heart indeed contributed to the embryonic CSF movement. The following work by Olstad et al. demonstrated that the zebrafish embryonic CSF circulation had two major components: a highly pulsatile flow located in the aqueducts and a directional flow along the ventricular wall [21]. The pulsatile component of the flow was strongest at the ducts and the center of the diencephalic ventricle. Additionally, they did not observe any net directional CSF flow across the brain ventricles when the animal is immobile. Motile cilia were the major contributor to compartmentalized CSF flow, whereas bodily movements, along with cardiac forces, drive the exchange of CSF among the brain ventricles. Intercompartmental CSF exchange was noted only with movement. They concluded that components of the ventricular CSF flow were only partially dependent on cardiac forces. Despite the lack of its significant contribution to the CSF movement, the pulsatile flow was demonstrated in the zebrafish ventricular space [21, 22].

In *Xenopus*, our findings, although agreed with the cilia's importance as the main driver of the embryonic CSF circulation, were fundamentally different. We observed no cardiac forces' contributions, no pulsatile flow, in contrast to the larval zebrafish model during early embryogenesis. Instead, intra and interventricular mixing across the entire ventricular system was solely driven by *Xenopus's* ciliary network. Until the active feeding stages (Stage 45), *Xenopus* remains mostly dormant; therefore, the contribution of bodily movement is minimal during the polarization of CSF circulation. On average, over 5 min, stage-37/38 animals swim at 0.4%, stage-43 3.11% and stage-45 36% of the time [74]. Beyond stage 46, *Xenopus* tadpoles swim with a relatively constant frequency, and the amplitude of the head oscillations is rather variable and decreases as tadpoles grow [75]. We did not detect changes in the CSF circulation pattern when we partially immobilized *Xenopus'* head and liberalized bodily movements (Additional file 16: Movie S12). Therefore, unlike the zebrafish, intercompartmental bi-directional CSF mixing relied on the ciliary network.

Finally, we showed the impact of CSF flow on thalamic patterning. The thalamus is a critical diencephalic structure situated on each side of the 3rd ventricle where sensory information is relayed. Based on the prosomeric model, the diencephalon is subdivided into well-conserved anteroposterior segments across species [76] (*Xenopus* [77], chick [78], zebrafish [79], mouse [80]), where LIM-homeodomain factors show alternating expression along the diencephalon and define embryonic subdivisions [80]. When we halted CSF circulation, we observed a dramatic loss of the thalamic marker *lhx1,* specifically in the telencephalic region. This change was in conjunction with the mis-regulation of the dorsal telencephalic marker *emx1*, which expanded well beyond the telencephalon. The change in expression of these two markers in the telencephalon may reflect a disruption in normal neuroprogenitor patterning due to a loss of CSF flow.

Interestingly, despite the changes in the prosencephalon, the impact of the absence of CSF flow on the midbrain and hindbrain was minimal. However, CSF flow speed is highest in these regions, suggesting that CSF circulation regionally may be interpreted differently. Together, these observations are in line with the long-standing notion that a connection exists between CSF circulation and neurogenesis. However, here, we establish another link and suggest that compartmental flow driven by the cilia contributes to “molecular boundaries.” The frog, as well as other semi-transparent animal model systems (e.g., zebrafish), in which CSF fluid dynamics can be easily coupled with molecular profiling using OCT imaging, will enable future studies to understand this complex interaction.

## Conclusion

Altogether, our study demonstrates that proper embryonic CSF circulation and subsequently, CNS development goes through stages of development where the ciliary flow is the sole driver before the emergence of pulsatile forces. At this critical phase, CSF flow is compartmentalized and sets molecular boundaries to regulate proper prosencephalic regionalization, and when lost, can lead to aqueductal stenosis, a hallmark of human hydrocephalus. Further studies are needed to investigate the links between cilia, CSF circulation, and human disease states in which these processes may be compromised, such as infantile hydrocephalus. A better understanding of embryonic CSF homeostasis could uncover targets that could be modulated to ameliorate hydrocephalus and other conditions.

## Supplementary Information

**Additional file 1: Figure S1.** Xenopus tropicalis ventricular development map by OCT imaging. **(A)**Xenopus tropicalis tadpole ventricular system developmental map shown between the stages 18 to 46. Stereomicroscopy image presented with the corresponding mid-sagittal OCT imaging. **(B)** OCT images of the stage 49 tadpole from coronal and midsagittal plane. Anterior choroid plexus outlined with red circles and magnified view in red square. Posterior choroid plexus outlined with green circles and magnified view in green square. a: anterior; p: posterior; d: dorsal; v: ventral.

**Additional file 2: Movie S1.** Video showing OCT movies of the ventricular space in stage 18, 32, 39, 46 *Xenopus tropicalis* tadpoles. The initial CSF movement is at stage 32. Polarization and bulk movement of the intraventricular particles and CSF is visible at stage 39. Five mature and distinctly polarized flow fields are visible at stage 46.

**Additional file 3: Movie S2.** Video shows a stage 30 tadpole mid-sagittal plane before and after the injection of the polystyrene microbeads. Beads remain static, no CSF movement visible (30fps).

**Additional file 4: Movie S3.** Video shows a stage 49 tadpole mid-sagittal plane before and after the injection of the polystyrene microbeads. Within the compartmental and intercompartmental space, the CSF movement is polarized. Intercompartmental CSF movement is bidirectional, dorsal flow directed to the rostral region, whereas ventral flow is directed caudally (30fps).

**Additional file 5: Movie S4.** Video is focused at the lateral and 3rd ventricle of the stage 49 tadpole. Intercompartmental CSF movement is bidirectional. Dorsal—rostral CSF flow from the third ventricle is opposite to the dorsal- caudal flow generated from the lateral ventricle. Similarly, ventral flow is also polarized in the opposite direction, 3rd ventricle flow is now caudal, whereas the lateral ventricle flow is directed rostrally (30fps).

**Additional file 6: Movie S5.** Video shows a stage 49 tadpole mid-sagittal plane focused at the 3rd, midbrain, and fourth ventricles. CSF movement is bidirectional and polarized along the rostral-caudal axis (30fps).

**Additional file 7: Movie S6.** Video shows a stage 49 tadpole mid-sagittal plane focused on 4th ventricle choroid plexus before and after NiCl2 injection (30fps).

**Additional file 8: Movie S7.** Video shows a stage 49 tadpole mid-sagittal plane focused on 4th ventricle choroid plexus after microbead injection followed by before and after NiCl2 injections.

**Additional file 9: Figure S3.** Ventricular Volume Measurements at Stage 46 tadpole: Control, Heart Ablation, c21orf49 morphant. Data was analyzed using Prism8 statistical software. Significance was determined when the p value is lower than 0.01. For comparison between controls, heart ablated tadpoles and *c21orf59* morphants we utilized Mann–Whitney test (nonparametric, unpaired) and used scatter plot graph where we show the mean with SEM. Significance was determined when the p value is lower than 0.01. (*p < 0.01, **p < 0.001, ***p < 0.001).

**Additional file 10: Movie S8.** Video shows a stage 46 tadpole ventricular system, CSF circulation, and the cardinal vein with the temporal color map at pre (left) and post (right) cardiac ablation states. Systemic cardiovascular circulation abolishes post cardiac ablation, whereas CSF circulation, ventricular morphology, and size remain unchanged.

**Additional file 11: Movie S9.** Video shows a stage 49 tadpole mid-sagittal plane focused on the hypothalamic region following NiCl2 injection. Pulsatile flow pointed with the white arrow.

**Additional file 12: Movie S10.** Video shows a stage 49 tadpole before and after Na2VO4 injection. The last movie is following multiple washes where ciliary movement is partially restored. The right column shows the corresponding temporal heat maps of 100frames (30fps).

**Additional file 13: Movie S11.** Video shows a stage 49 tadpole before and after nickel chloride hexahydrate injection. The last movie is following multiple washes where ciliary movement is partially restored (30fps).

**Additional file 14: Figure S2.** Stage 46—*Xenopus laevis* ventricular cilia distribution in whole brain. Fluorescence images of **(A)** the most lateral and **(A’)** mid-sagittal view of the whole brain. Cilia (green) marked with anti-GT335 antibody. **(B)** Fluorescence image of the dorsal view the whole brain shows clusters of MCCs along the dorsal lateral, third and fourth ventricles. **(B1)** Anterior choroid plexus, **(B2)** pineal gland region, **(B3)** posterior choroid plexus, magnified views of the representative regions. R: right, L: left, a: anterior; p: posterior; d: dorsal; v: ventral.

**Additional file 15: Figure S4.** *emx1, lhx1* and *en2* expression in controls, *c21orf59* morphants and heartless tadpoles. Quantification of **(A, D)** *emx1*, **(B, E)** *lhx1*, **(C, F)** *en2* mRNA expression in stage 46 control vs. *c21orf59* morphant tadpoles **(A-C)** and control vs. heart ablated tadpoles **(D-F).** *emx1* expression was categorized as dorsal telencephalon only or dorsal telencephalon + diencephalon extension. *lhx1* expression was categorized as telencephalon positive or negative. *en2* expression was categorized as mid-brain hindbrain border positive or negative. UIC: un-injected control, HA: heart ablated.

**Additional file 16: Movie S12.** Left video shows freely swimming stage46 tadpole, where the distal tail mostly drives bodily movements. Similarly, the middle video shows the partially immobilized stage 46 tadpole, where the tail remains free. The OCT video is obtained from the partially immobilized tadpole during free tail movements (30fps).

## Data Availability

The data that support the findings of this study are available from the corresponding author upon reasonable request.

## Abbreviations

CSF: Cerebrospinal fluid
OCT: Optical coherence tomography
CNS: Central nervous system
MCCs: Multiciliated cells
FF: Flow fields
Zli: Zona limitans intrathalamica
