# Supplementary figures and images for: In Xenopus ependymal cilia drive embryonic CSF circulation and brain development independently of cardiac pulsatile forces
Source: Fluids Barriers CNS. 2020 Dec 11;17:72. doi: 10.1186/s12987-020-00234-z (PMC7731788; doi:10.1186/s12987-020-00234-z)

Mid-sagittal plane

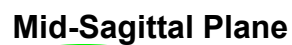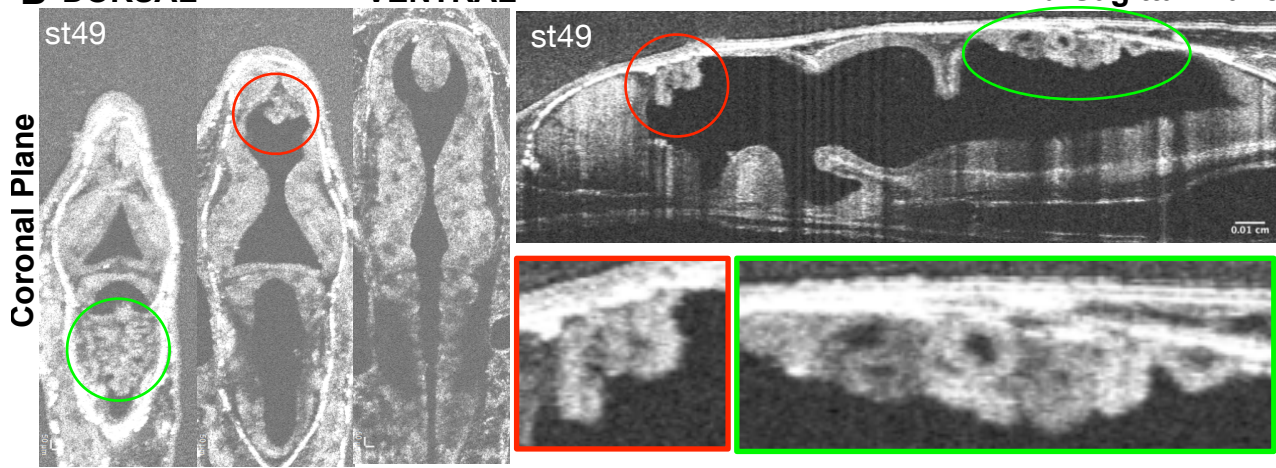

Supplement: Supplementary file 1 — Additional file 1: Figure S1. Xenopus tropicalis ventricular development map by OCT imaging. (A)Xenopus tropicalis tadpole ventricular system developmental map shown between the stages 18 to 46. Stereomicroscopy image presented with the corresponding mid-sagittal OCT imaging. (B) OCT images of the stage 49 tadpole from coronal and midsagittal plane. Anterior choroid plexus outlined with red circles and magnified view in red square. Posterior choroid plexus outlined with green circles and magnified view in green square. a: anterior; p: posterior; d: dorsal; v: ventral. [file 12987_2020_234_MOESM1_ESM.pdf]

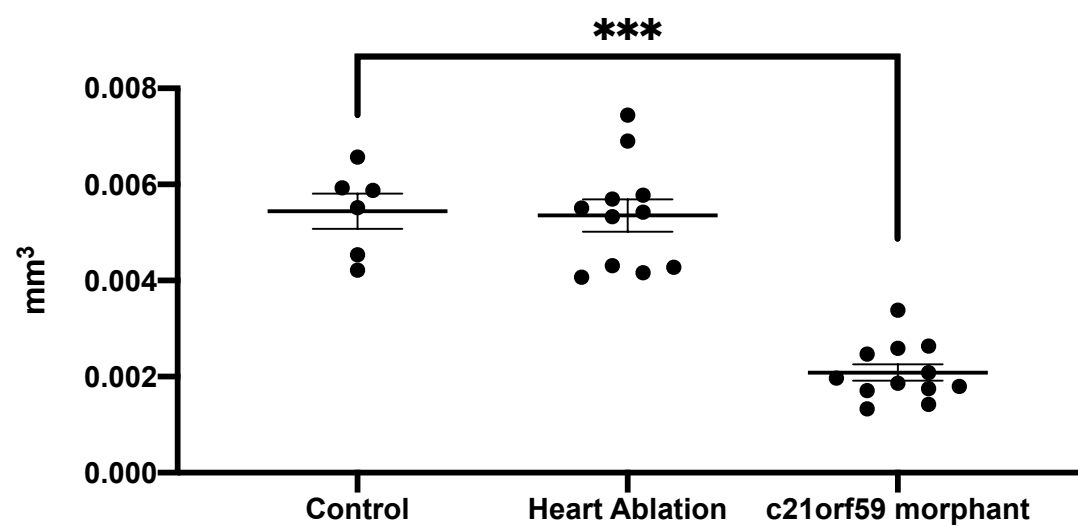

Supplement: Supplementary file 9 — Additional file 9: Figure S3. Ventricular Volume Measurements at Stage 46 tadpole: Control, Heart Ablation, c21orf49 morphant. Data was analyzed using Prism8 statistical software. Significance was determined when the p value is lower than 0.01. For comparison between controls, heart ablated tadpoles and c21orf59 morphants we utilized Mann–Whitney test (nonparametric, unpaired) and used scatter plot graph where we show the mean with SEM. Significance was determined when the p value is lower than 0.01. (*p < 0.01, **p < 0.001, ***p < 0.001). [file 12987_2020_234_MOESM9_ESM.pdf]

Stage 46 *Xenopus laevis*

A

GT355

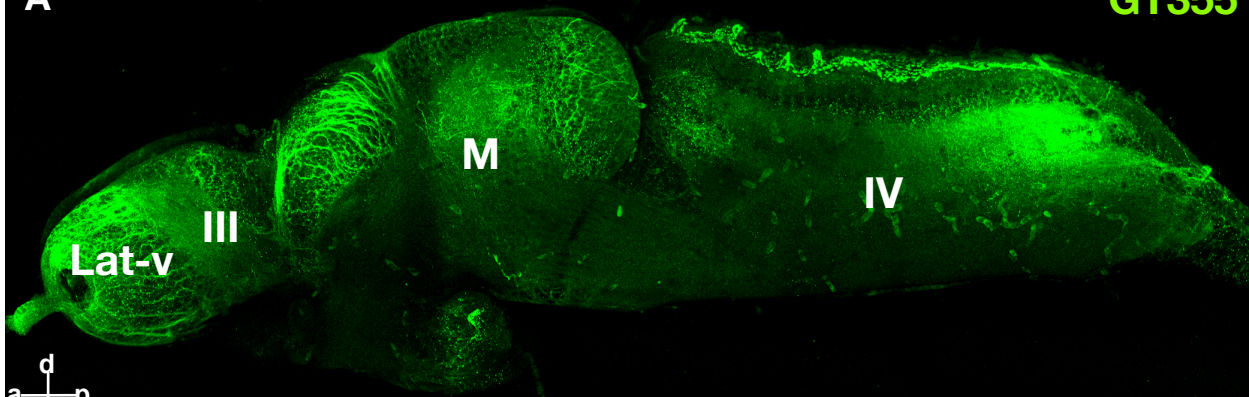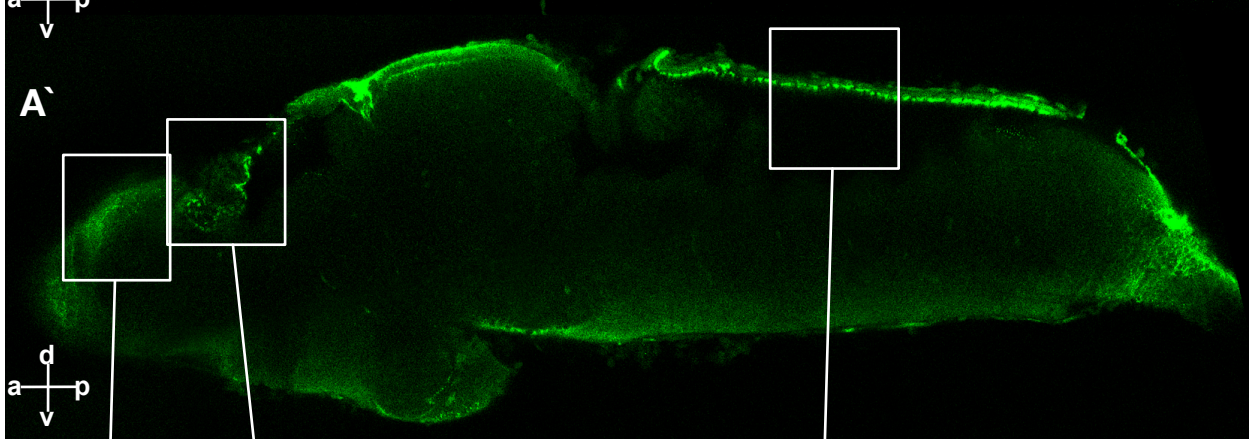

B

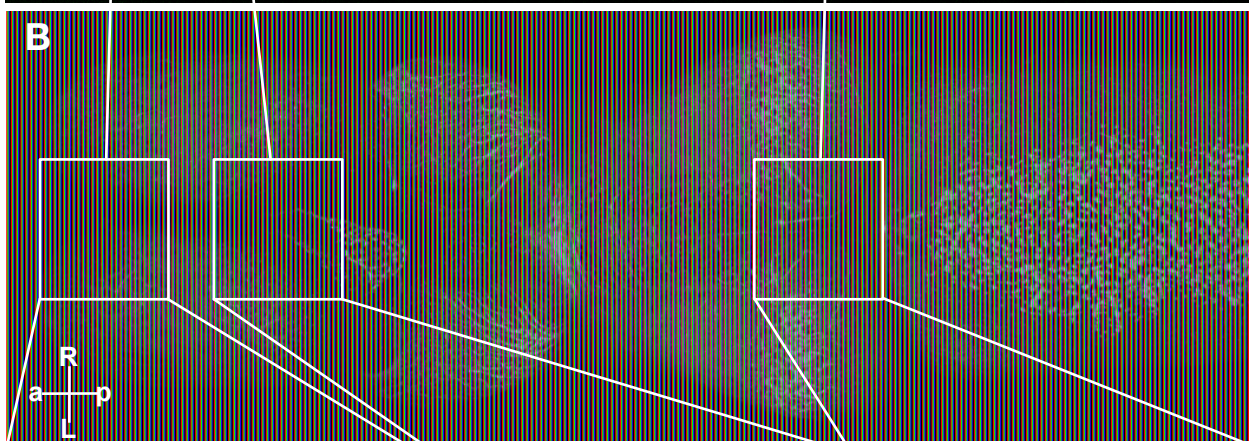

(B1)

(B2)

(B3)

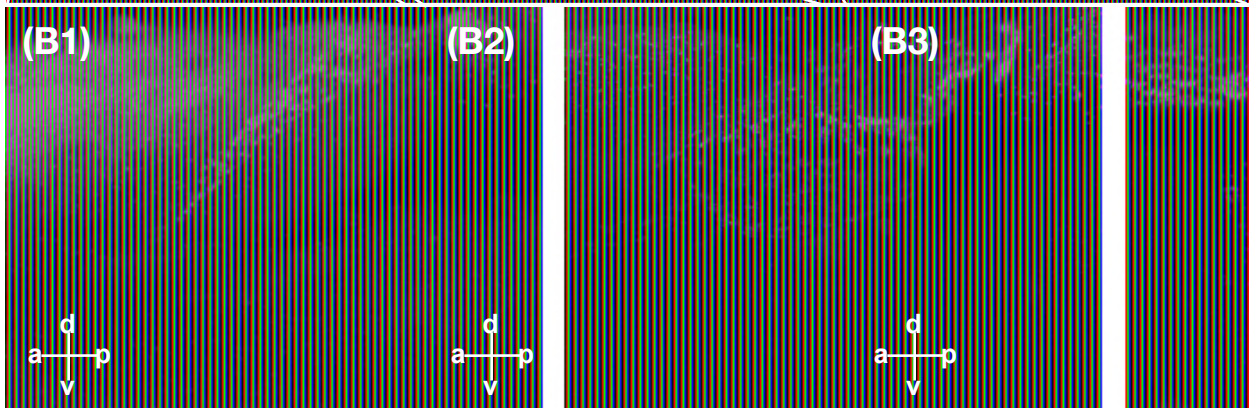

Supplement: Supplementary file 14 — Additional file 14: Figure S2. Stage 46—Xenopus laevis ventricular cilia distribution in whole brain. Fluorescence images of (A) the most lateral and (A’) mid-sagittal view of the whole brain. Cilia (green) marked with anti-GT335 antibody. (B) Fluorescence image of the dorsal view the whole brain shows clusters of MCCs along the dorsal lateral, third and fourth ventricles. (B1) Anterior choroid plexus, (B2) pineal gland region, (B3) posterior choroid plexus, magnified views of the representative regions. R: right, L: left, a: anterior; p: posterior; d: dorsal; v: ventral. [file 12987_2020_234_MOESM14_ESM.pdf]

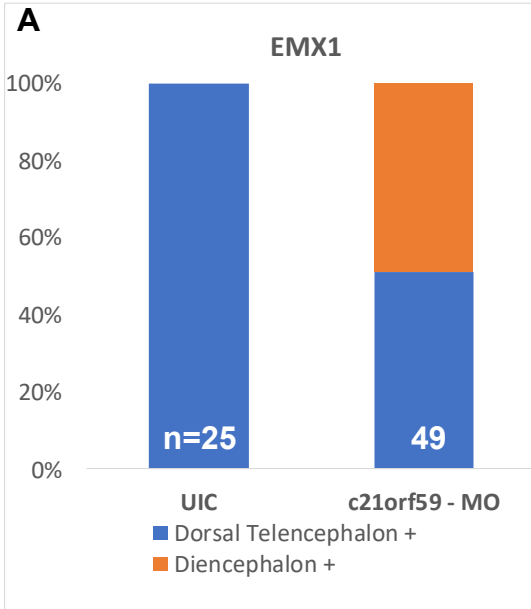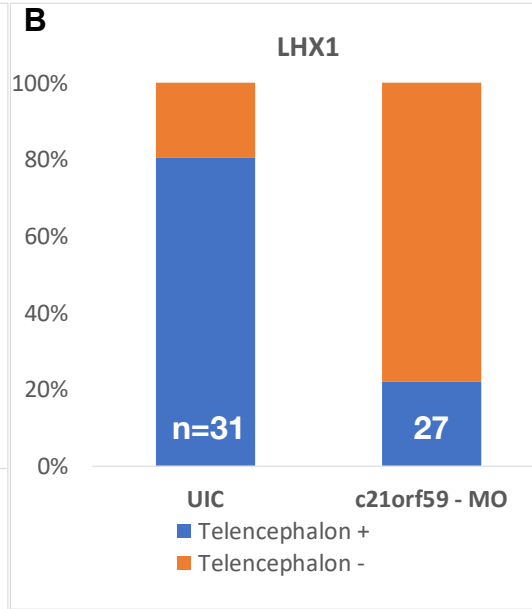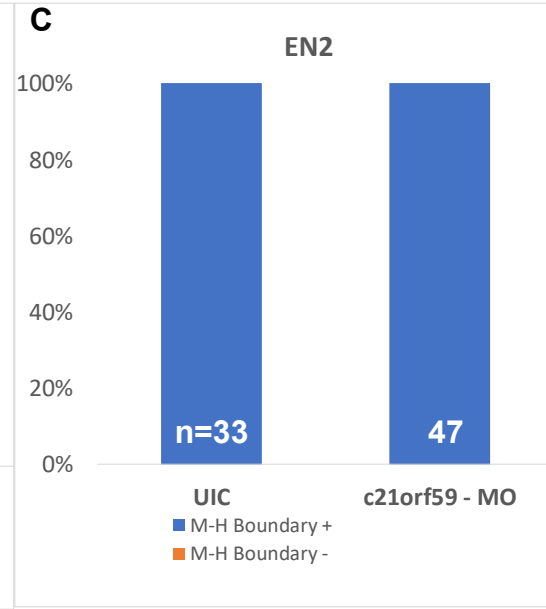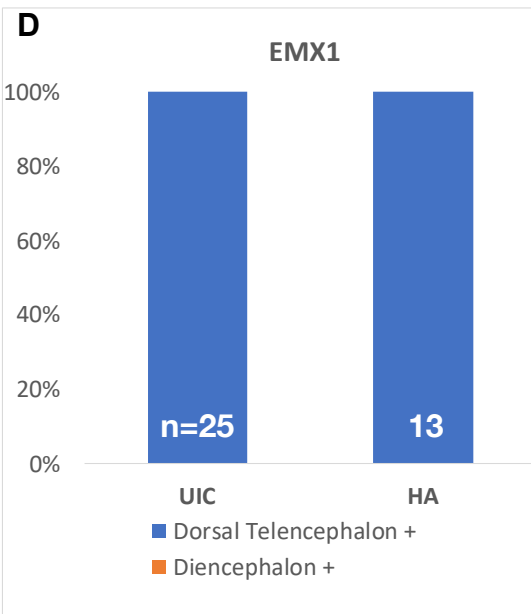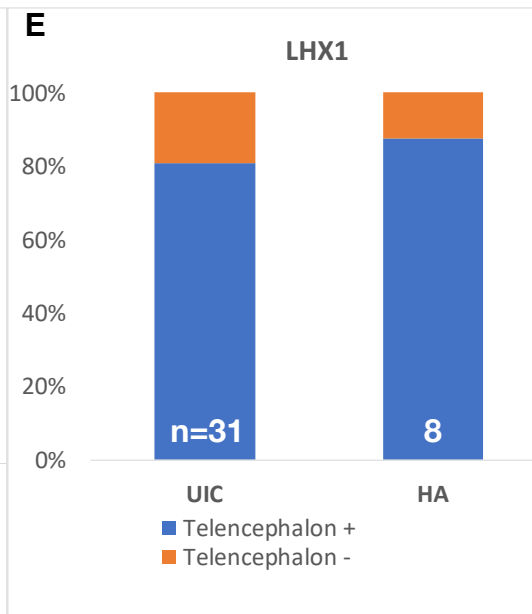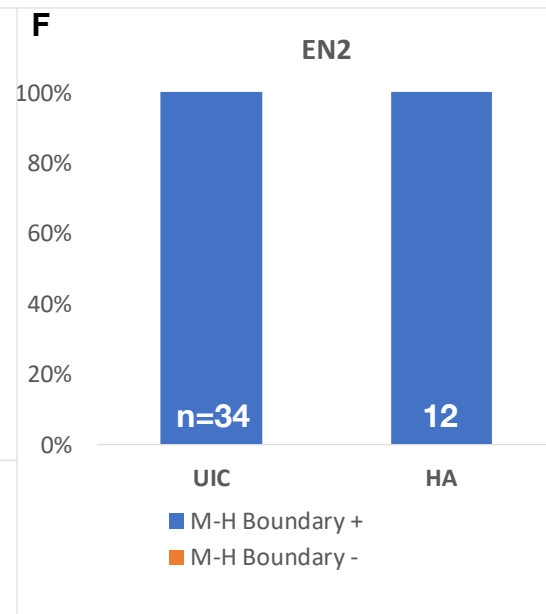

Supplement: Supplementary file 15 — Additional file 15: Figure S4. emx1, lhx1 and en2 expression in controls, c21orf59 morphants and heartless tadpoles. Quantification of (A, D) emx1, (B, E) lhx1, (C, F) en2 mRNA expression in stage 46 control vs. c21orf59 morphant tadpoles (A-C) and control vs. heart ablated tadpoles (D-F). emx1 expression was categorized as dorsal telencephalon only or dorsal telencephalon + diencephalon extension. lhx1 expression was categorized as telencephalon positive or negative. en2 expression was categorized as mid-brain hindbrain border positive or negative. UIC: un-injected control, HA: heart ablated. [file 12987_2020_234_MOESM15_ESM.pdf]
